# Dipping and rotating: two maneuvers to achieve maximum magnification during indirect transnasal laryngoscopy

**DOI:** 10.1007/s00405-020-05862-7

**Published:** 2020-03-04

**Authors:** Susanne Fleischer, Christina Pflug, Markus Hess

**Affiliations:** 1 Department of Voice, Speech and Hearing Disorders, University Medical Center Hamburg-Eppendorf, Martinistrasse 52, 20246 Hamburg, Germany; 2 Deutsche Stimmklinik (German Voice Clinic), Martinistrasse 64, 20251 Hamburg, Germany

**Keywords:** Flexible laryngoscopy, Rotation laryngoscopy, Dipping maneuver, Magnification, Zoom laryngoscopy

## Abstract

**Background:**

Since many years, office-based flexible transnasal laryngoscopy is a common routine procedure. The development of new technical equipment such as high-definition cameras and flexible tip-chip endoscopes nowadays allows for much more precise examination than a few years ago. In contrast to rigid laryngoscopy, it is possible to move the tip of the endoscope close to the vocal folds and to other structures of interest. Nevertheless, without professional handling of the equipment, one cannot benefit from the potential of the newest technology.

**Method:**

Two easily performed and very helpful maneuvers in flexible endoscopy are described. The “dipping maneuver” enables a maximum magnification of the mucosal surfaces of the endolarynx as well as the examination of the subglottal region and the trachea by positioning the tip of the endoscope very close to the vocal folds or even in the upper trachea during long transnasal inspiration. During the “rotation laryngoscopy”, the tip of the endoscope is positioned in the posterior interarytenoid region by rotating the flexible endoscope by 180° and advancing it close to the glottis. This allows a close-up examination of the anterior commissure, the inferior aspect of the vocal folds and the inside of the Morgagni’s ventricle. Before performing transnasal flexible endoscopy, we routinely apply topical anesthesia sprayed intranasally.

**Conclusion:**

The described techniques of flexible endoscopy are easily performed and allow a maximum magnification of the mucosal surfaces and otherwise not visible regions of the endolarynx.

## Introduction

One important feature in office-based endoscopy is the fact that when the lens gets closer to the target, the image resolution is enhanced. This is even more true for small endoscopes with tiny lenses. Here, millimeters in distance can make a big difference. We describe two easily performed clinical handling maneuvers in flexible endoscopy which enable a much better magnification of the mucosal surfaces of the endolarynx. These maneuvers allow a close-to-target imaging, thus gaining an extraordinary magnification and quite astonishing 'insights' within office-based endoscopy.

## Materials and methods

We demonstrate two techniques we routinely use for highest image resolution in flexible transnasal laryngoscopy.

### Materials

For transnasal flexible endoscopy, we commonly use the flexible video endoscope type ENT-VH with the image processing system EVIS EXERA III CV-190 (Olympus Medical Systems Corp., Tokyo, Japan). The outer diameter of the distal end of this endoscope is 3.9 mm. Both maneuvers can be performed with endoscopes with smaller or larger diameters as well. For children, we use endoscope type ENT-V3 with a diameter of 2.6 mm. For the endoscope with a working channel (type ENT-VT2), we use a diameter of 4.9 mm. All videos are recorded with Black Magic (compression set to 20.0 mbps) on a Mac (Type: iMac). Off-line image analysis is achieved with QuickTime Player. We use this setting for all our patients.

### Methods

Before flexible endoscopy, we routinely apply topical anesthesia (lidocaine 4%, approx. 1 ml) sprayed during nasal inhalation to numb nasal cavity, nasopharynx and mesopharynx. In patients with pronounced gag response and in cases with indication for transnasal tracheoscopy (TNT), we additionally spray or instill anesthesia (lidocaine 4%, approx. 1 ml) transorally into hypopharynx and endolarynx (‘laryngeal gargle’). The average length of our endoscopic evaluation with the entire endoscopy is approx. 5–10 min and these special maneuvers take about 2–3 min. We estimate that 95% of our patients can tolerate the maneuvers, if not more, with the routinely administered topical anesthesia.

#### Technique I: endolaryngeal dipping maneuver during flexible endoscopy

**Fig. 1:**
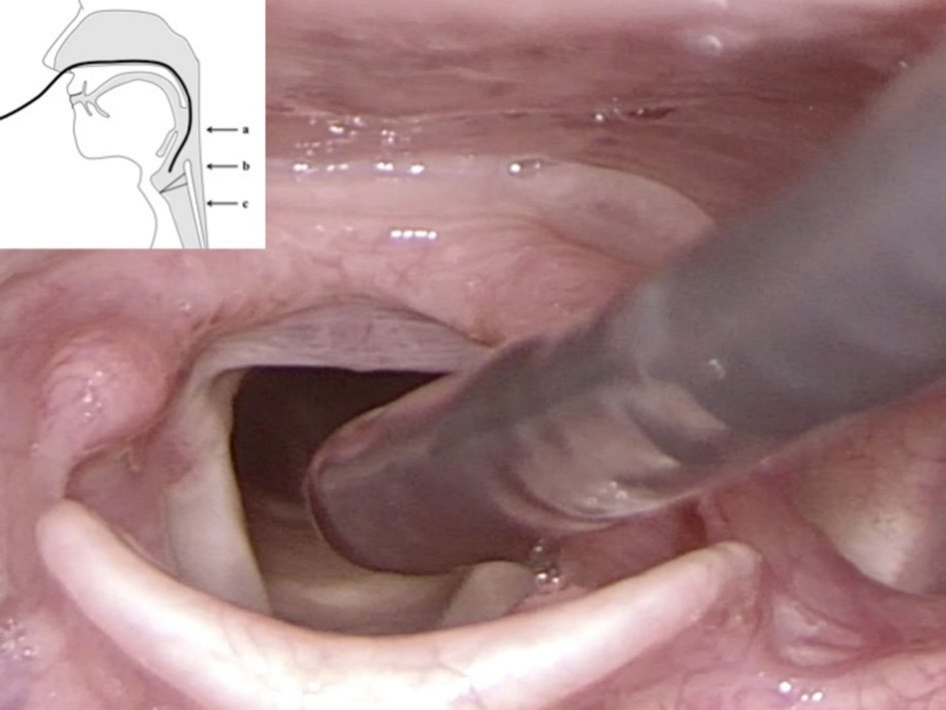
Rigid transoral endoscopy visualizes tip of flexible endoscope during “dipping” into the endolarynx. Picture in picture: scheme showing the different positions of the tip of the endoscope. **a** standard level of tip of endoscope, **b** position very near to the vocal fold, **c** endotracheal position

The ‘dipping maneuver’ denotes a short maneuver where the flexible endoscope is advanced into and retracted from the endolarynx during a maximally prolonged, voluntary inspiration through the nose. At the beginning of a many seconds lasting inspiration phase, the endoscope gets very close to the vocal folds with closest distance at mid-inspiration. The surface is almost contacted in maximum ‘dipping in’. Before the end-expiratory phase is reached, the endoscope is rapidly withdrawn to avoid any touching of the (gag-eliciting) medial surface of the arytenoids. The whole maneuver resembles a ‘dipping’ of the endoscope into the larynx (Fig. 1).

We recommend to verbally instruct and guide the patient, so he can better tolerate the procedure. Prior to performing the dipping maneuver, it is helpful first to practice the long transnasal inspiration with the patient. A transnasal inspiration serves two purposes: first, transnasal inspiration is a very strong vocal fold abductor stimulus. Second, high inspiration resistance prolongs inspiration. With this test run, the patient learns to prolong a steady transnasal inspiration, and the endoscopist becomes acquainted with the patient’s individual gag tolerance. One should avoid triggering gag and cough reflexes by touching any laryngeal structures. To achieve an even longer inspiratory phase, it can be helpful to increase the inspiratory resistance by closing the free nasal inlet (the side not being occupied with the endoscope) by the examiner. Alternatively, the patient can close the nose himself.

#### Technique II: rotation laryngoscopy in flexible endoscopy

**Fig. 2:**
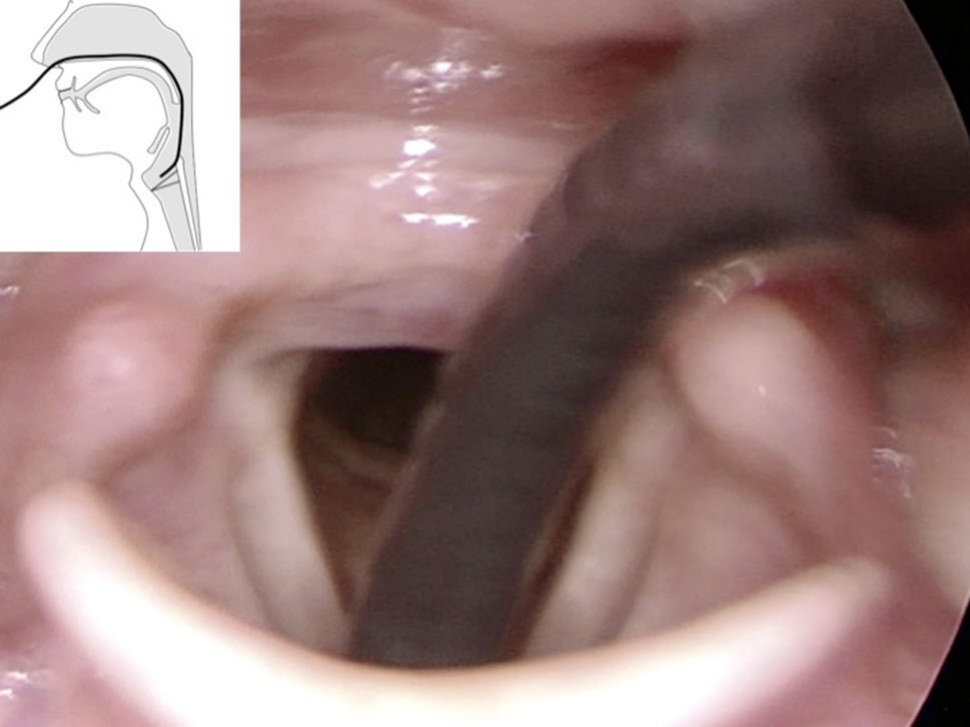
Rigid transoral endoscopy visualizes rotated flexible endoscope with the tip more horizontal compared to Fig. 1. Picture in picture: scheme showing the tip of the endoscope during rotation with endoscope slightly more rounded and tip more horizontal

**Fig. 3:**
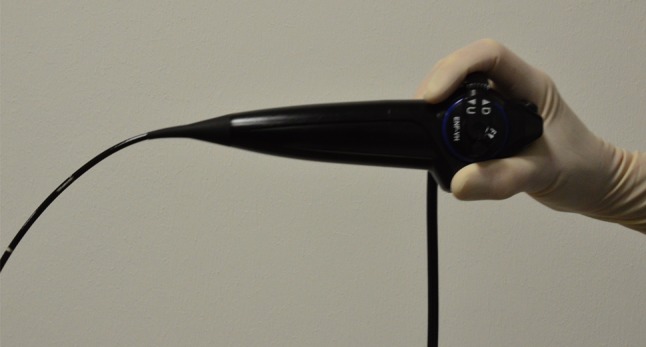
Endoscope held in standard position

**Fig. 4:**
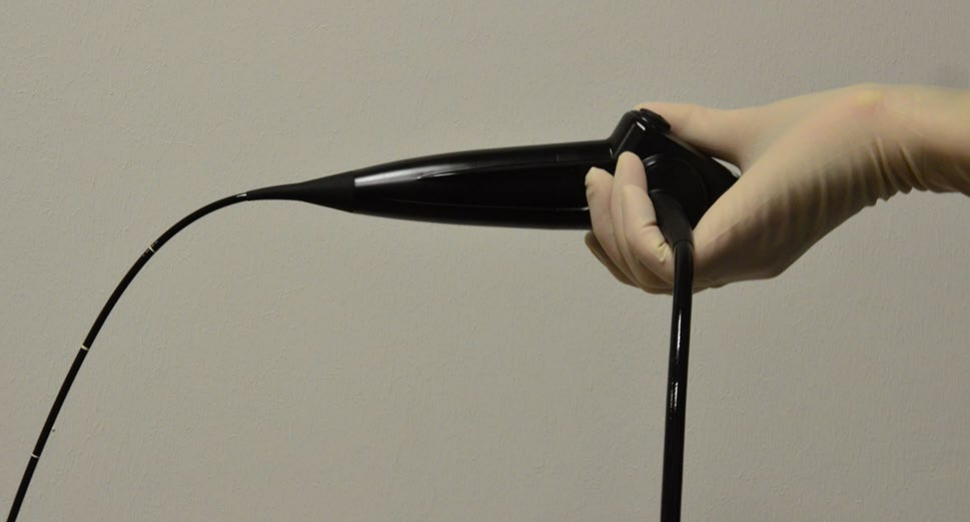
Rotation of endoscope

After inserting the flexible endoscope transnasally in the normal manner with the tip at the level of the uvula, then, by rotating the flexible endoscope by 180°, the tip of the endoscope will still be positioned in the posterior mesopharyngeal region. By advancing the endoscope slowly into the endolarynx, the endoscope tip will approach close to the vocal folds and the anterior commissure in a more horizontal posterior-to-anterior vector (Figs. 2, 3, 4). This allows examination of the anterior commissure as well as the medial or inferior aspect of the vocal folds, Morgagni’s ventricle, and the subglottal region.

## Results

### Technique I: dipping maneuver

**Fig. 5:**
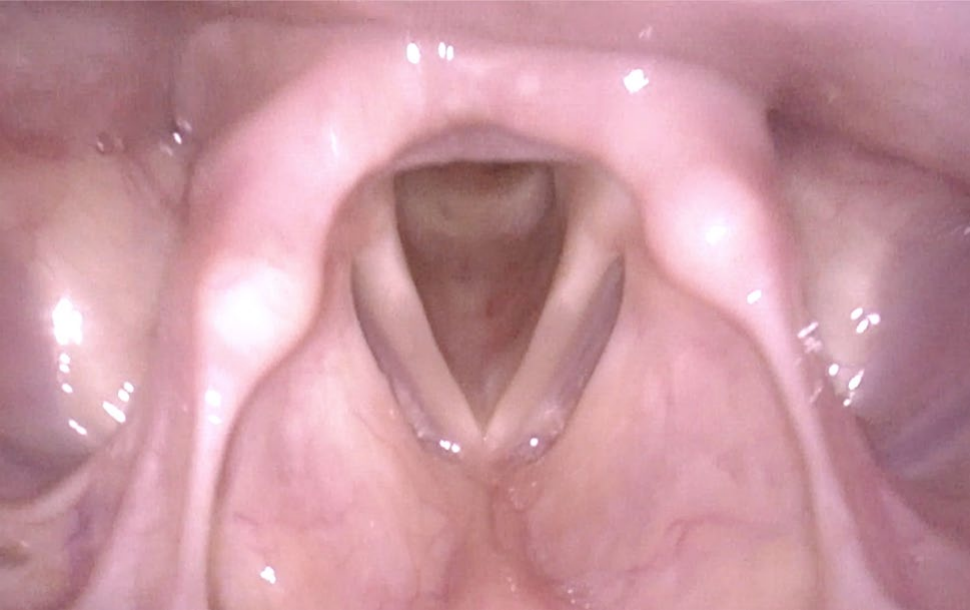
Normal larynx, seen during transnasal endoscopy with standard distance between the tip of endoscope and vocal folds

**Fig. 6:**
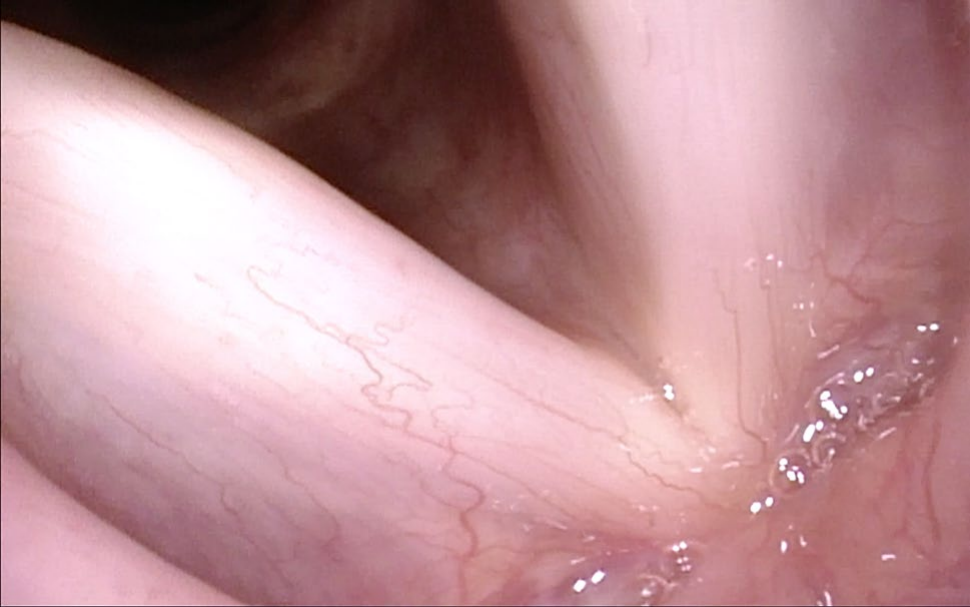
Normal larynx, seen with the tip of the endoscope very near to the vocal fold

By advancing the tip of the flexible endoscope very close to the glottis, magnification of the vocal folds is much better than in images obtained with the routinely used distance from the superior edge of the epiglottis to the vocal folds (Figs. 5, 6). In most cases, also an insight into the trachea is possible.

### Technique II: rotation laryngoscopy

**Fig. 7:**
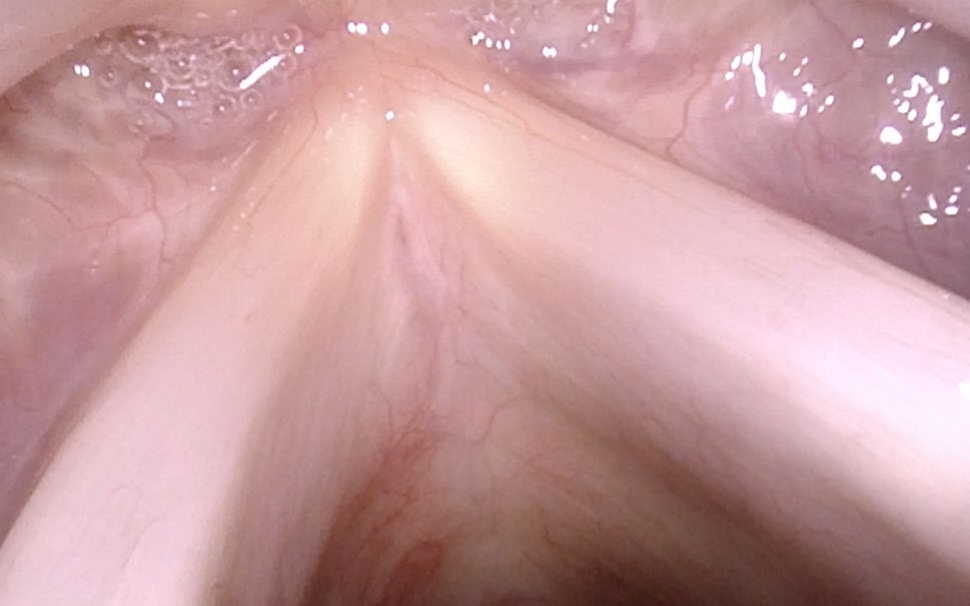
Normal larynx, seen during rotation maneuver. Good visible inferior aspect especially of the right vocal fold and the anterior commissure

**Fig. 8:**
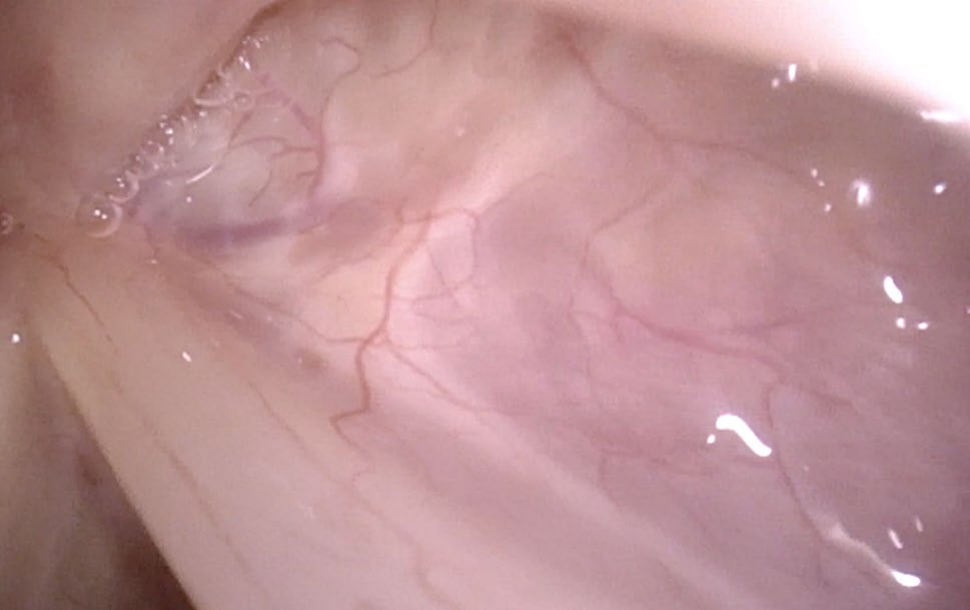
Normal larynx, seen during rotation maneuver with good view into anterior right sinus Morgagni

**Fig. 9:**
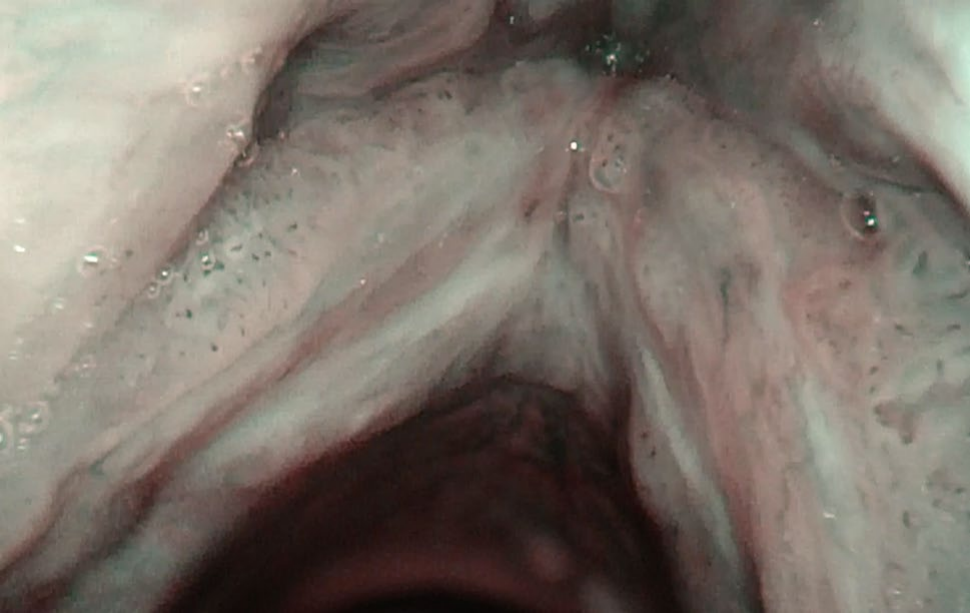
Anterior commissure, seen with the rotation maneuver under NBI in a patient with laryngeal papillomatosis. In Figs. 6, 7, 8 and 9, magnification was achieved by approaching the tip of the endoscope very close to the target. No optical or digital magnification was used

With the oblique posterior-to-anterior access, the anterior commissure can be seen completely even with its inferior part, the inferior surfaces of the vocal folds are made visible, and Morgagni’s ventricle can be inspected (Figs. 7, 8, 9).

## Discussion

Office-based flexible transnasal laryngoscopy is a common routine procedure since many years. The development of new technical equipment such as flexible high-definition endoscopes allows for very precise examination [1–9]. The basic principle for getting optimal magnification in laryngoscopy is to decrease the distance of the lens to the target, i.e., the nearer one gets to the vocal fold, the more details one can see. This is especially important for transnasal flexible laryngoscopy because of the small lenses with wide aperture angles. Very small lesions and fine structures of the vocal folds can be evaluated best when the tip of the endoscope is advanced very close-to-target and almost touching the vocal folds. Tiny but diagnostically significant lesions can be missed with insufficient magnification.

For assessing the resolution of the imaging system one is working with, it is helpful to use a device that can demonstrate its possibilities and limitations. For this purpose, we use a special endoscope calibration cube (“Eppendorf cube” provided by Olympus, [10]), but a graphic with mm measures would help as well.

The advantage of flexible endoscopy, compared with rigid endoscopy, is the possibility to move the tip of the endoscope more easily into the endolarynx. Especially, the dipping maneuver with positioning the tip of the endoscope very close to the vocal folds is an easily performed maneuver in routine flexible endoscopy [10]. Our experience shows that this close-up maneuver is most easily performed during long nasal inspiration—here, the vocal folds normally abduct maximally. By increasing the nasal airway resistance by closing one nasal inlet, inspiration can be intentionally prolonged with high inspiratory airflow resistance due to an overall small nasal lumen. For successful performance of the dipping maneuver, one should avoid eliciting a gag response by touching sensitive laryngeal structures. Mostly it is advisable for patient’s compliance to first do some trial maneuvers without endoscope dipping to get the patient used to the procedure [11].

Still, even with best compliance during the dipping maneuver, some structures cannot be seen when the tip of the endoscope advances towards the vocal folds from this vertical direction. Here, another helpful maneuver in flexible endoscopy can be applied, the ‘rotation laryngoscopy’. It is a well-established, however, rarely used technique. Although we could not find any literature describing its first publication, we acknowledge the colleagues who invented this technique. The rotation laryngoscopy allows examination of some endolaryngeal regions of interest one would otherwise not see, especially the anterior commissure with its inferior part, the inferior aspect of the vocal folds, Morgagni’s ventricles, and the subglottic region. Here, in our experience, even slight changes of the angle of the tip of the endoscope can be extremely helpful. When performing office-based surgery in local anesthesia with a flexible channelled endoscope, e.g., for injections or laser surgery, access to these regions is often more easy with rotation laryngoscopy. The rotation laryngoscopy can also be combined with the dipping maneuver for optimal laryngeal exposure of the region of interest.

To avoid camera image overmodulation and “washout” under zoom conditions, sometimes it is advisable to switch between camera settings (automatic gain control (AGC) versus manual gain control), or to use dimmed light, or to change to another illumination-like (mostly less bright) stroboscopy light or narrow band imaging (NBI).

In rigid laryngoscopy, resolution is often better due to better optical systems, but further enhancement by decreasing the distance between lens and target for getting optimal magnification might be a problem. In some cases, but not in all, this can be achieved by lowering the tip of the rigid endoscope as near to the laryngeal inlet as possible by pressing down the base of the tongue.

The described maneuvers can be performed in nearly all patients—in some cases, additional anesthesia might be helpful. We use these maneuvers routinely and never had any complications such as severe vasovagal reactions or laryngospasm.

## Conclusion

For achieving an optimal image magnification (and, therefore, resolution) in flexible laryngoscopy, one has to decrease the distance of the lens to the target, due to the fact that the closer one gets to the vocal fold, the more details one can see. The combination of dipping maneuver and rotation laryngoscopy enables the examiner to accomplish two targets: enhanced image resolution and visualisation of otherwise not accessible parts of the endolarynx such as the anterior commissure with its inferior part, the medial and inferior aspect of the vocal folds with the subglottic region, Morgagni’s ventricles and sometimes the trachea as well. In our clinical practice, both maneuvers are routine procedures since many years.
